# Cell–Cell Communication and Extracellular Vesicles in Cancer

**DOI:** 10.3390/cancers15092419

**Published:** 2023-04-23

**Authors:** Ling Lin, Yandong Zhou, Kebin Hu

**Affiliations:** 1 Department of Medicine, Penn State University College of Medicine, Hershey, PA 17033, USA; 2 Department of Cellular and Molecular Physiology, Penn State University College of Medicine, Hershey, PA 17033, USA; yzhou1@pennstatehealth.psu.edu

Cell–cell communication, either through direct contact or indirectly, is critical for multiple cellular processes, such as proliferation, survival, differentiation, and transdifferentiation, and it plays a fundamental role in maintaining the integrity of tissue structure and cellular environment. Extracellular vesicles (EVs) are a group of nanosized lipid-bound vesicles derived from various cellular origins, and they are secreted by all cell types and organisms. Although EVs have been subcategorized into several subgroups based on their biogenesis pathway and size, the most common two subsets are exosomes and microvesicles (MVs, also called ectosomes or microparticles) [1,2]. The diameter size of exosomes is between 30 and 150 nm, while that of MVs is usually within the range of 50–1000 nm. Exosomes, containing various biomolecules such as proteins, RNAs, DNAs, lipids, and metabolites, are formed through an endocytic pathway which results in the formation of intraluminal vesicles by inward budding of the multivesicular bodies [2], whereas MVs are formed through outward budding of plasma membrane [1]. EVs contain various cell type-specific cargos that play a key role in cell–cell communication. EV-mediated cell–cell communication participates in multiple processes of tumorigenesis and metastasis. It not only affects tumor growth, metabolism, and survival, but also facilitates the surrounding or distant non-tumor cells to form a protumor microenvironment. Notably, recent studies indicate that EVs have the potential to serve as a next-generation drug delivery platform for cancer treatment.

In this Special Issue, seven quality manuscripts were selected for publication covering a relatively broad topic of intercellular communication and EVs in cancers. Three of these publications are review articles reflect the current knowledge and advances in the understanding of cell–cell communication and EVs in carcinogenesis. A review article from Chen HJ group was curated as Editor’s Choice and provided a comprehensive review regarding the energy sources for exosome communication in tumor microenvironment (TME) [3]. Exosomes play a critical role in both physiological and pathological conditions. Their involvement in cell–cell communication, including biogenesis, release or secretion, transportation to adjacent and distant sites, and eventually incorporation into the specifically targeted cells, is driven by various sources of energy that are currently largely unknown. Chen HJ group discussed various exosomal constituents that may serve as sources of energy to overcome the energy barriers for exosomal secretion and engagement in intercellular communication in TME, including surface charge, i.e., zeta potential, in exosomal function and behavior, long distance and site-specific transport of exosomes, bioenergetics of exosome release and uptake, perseverance of exosome structural integrity, interplay between exosomes and glycolytic metabolons, and ATP as an energy source for EVs in cancer progression. The authors also envisioned that reprogramming of exosomes to mimic cellular process may pave a way for artificial sources of energy that can be translated into new therapeutic approaches. Shan Y et al. reviewed the role of EVs in nasopharyngeal carcinoma (NPC) progression and therapeutic resistance with focus on the interaction of NPC cells and TME through EVs and the role of EVs in NPC radiation and chemoresistance [4]. They concluded that EVs have great potential to be biomarkers for NPC early diagnosis and therapeutic targets. Wang J and others highlighted the role of crosstalk between cancer cells and neurons in pancreatic cancer-associated perineural invasion and pain transmission [5]. They provided an important update regarding tumor–nerve interaction and underlying mechanisms of intercellular communication, although the role of EVs in such interactions remains to be elucidated in future.

Four of the publications in the collection of this Special Issue are original research articles with emphasis on EV-mediated intercellular communication in cancers. Andreucci E and others revealed that the release of tumor miR-214-enriched EV, potentiated by adapting tumor cells to extracellular acidosis, drives a macrophage-dependent trans-endothelial migration of melanoma cells, suggesting miR-214 as a potential new therapeutic target against melanoma intravasation [6]. Mantile F et al. illustrated a novel localization of EGF-CFC founder member CRIPTO in human large EVs using multiple analytic approaches such as Western blot, nanoparticle tracking analysis, and electron microscopy. Their findings provided mechanistic insights into EV-mediated negative regulation of glioblastoma cell migration [7].

Prostate cancer is one of the leading causes of cancer-related deaths lacking non-invasive specific biomarkers for early detection and diagnosis. Urine EVs could provide such markers; however, due to technical challenges, little is known regarding the pathogenic factors harbored in these EVs. Two of original research articles focused on this aspect: First, Puhka M group analyzed the miRNA cargo of urine EVs as a liquid prostate cancer biopsy from 31 prostate cancer patients (pre-prostatectomy) and compared miRNA expression based on cancer stage (Gleason Score) and progression (post-prostatectomy follow-up), determining that changes in miR-892a, miR-223-3p, and miR-146a-5p corelated with cancer stage and progression [8]. Their results illuminated the role of urine EV-containing miRNAs as potential biomarkers for prostate cancer diagnosis and monitoring. Second, Allelein S and colleagues developed a potential liquid biopsy strategy for prostate cancer diagnosis by enriching and measuring urine prostate-specific membrane antigen-positive EVs from 26 prostate cancer patients and 16 benign male individuals [9]. They developed a device with an adapted protocol that enables an automated immunomagnetic enrichment of EVs of large sample volumes (up to 10 mL) while simultaneously reducing the overall bead loss and hands-on time. This automated and specific enrichment of EVs from urine has a translational potential for future diagnostics.

In summary, these articles highlight the role and mechanism of EVs and cell–cell communication in various cancers and provide guidance for future research on these aspects, as well as development of specific and efficient treatments.
